# Draft Genomes for Eight *Burkholderia mallei* Isolates from Turkey

**DOI:** 10.1128/genomeA.01234-15

**Published:** 2016-01-07

**Authors:** H. E. Daligault, S. L. Johnson, K. W. Davenport, T. D. Minogue, K. A. Bishop-Lilly, S. M. Broomall, D. C. Bruce, S. R. Coyne, K. G. Frey, H. S. Gibbons, J. Jaissle, G. I. Koroleva, J. T. Ladner, C.-C. Lo, C. Munk, M. J. Wolcott, G. F. Palacios, C. L. Redden, C. N. Rosenzweig, M. B. Scholz, P. S. Chain

**Affiliations:** a Los Alamos National Laboratory (LANL), Los Alamos, New Mexico, USA; b United States Army Medical Research Institute of Infectious Diseases (USAMRIID), Fort Detrick, Maryland, USA; c Naval Medical Research Center (NMRC), Frederick, Fort Detrick, Maryland, USA; d Henry M. Jackson Foundation, Bethesda, Maryland, USA; e United States Army Edgewood Chemical Biological Center (ECBC), Aberdeen Proving Ground, Aberdeen, Maryland, USA; f Michigan State University (MSU), East Lansing, Michigan, USA

## Abstract

*Burkholderia mallei*, the etiologic agent of glanders, is a Gram-negative, nonmotile, facultative intracellular pathogen. Although glanders has been eradicated from many parts of the world, the threat of *B. mallei* being used as a weapon is very real. Here we present draft genome assemblies of 8 *Burkholderia mallei* strains that were isolated in Turkey.

## GENOME ANNOUNCEMENT

*Burkholderia mallei* is a host-adapted bacterium that is not known to persist outside of its equine reservoir. The organism causes the zoonosis glanders, which is endemic in Asia, Africa, the Middle East, and Central and South America. Infection by *B. mallei* typically occurs via the respiratory or percutaneous route, and the most common manifestations are life-threatening pneumonia and bacteremia. Glanders is difficult to diagnose and requires prolonged antibiotic therapy with low success rates. There is no vaccine to protect against *B. mallei* and there is concern regarding its use as a biothreat agent (1). Although human epidemics have not been recorded, isolated outbreaks in human populations and the deliberate use of *B. mallei* as a biological weapon have been documented (2, 3). In fact, *B. mallei* was one of the first biological warfare agents used in the 20th century, specifically during World War I (4). *B. mallei* has been considered a potential threat since the 1940s because of its high infectivity, degree of incapacitation, and agent availability. Here we present the draft genome sequences of 8 *B. mallei* strains that were collected in Turkey.

The *B. mallei* Turkey strains were isolated from either human or horse glanders cases (Table 1) by the Etlik Veterinary Control and Research Institute (Ankara, Turkey). We do not know the original isolation date for the horse isolates. The human isolates were obtained in the 1960s. The isolates were passaged in guinea pigs every 2 to 3 years to maintain pathogenicity and were re-isolated from the spleen and liver. The last passages of these isolates were in January 1984. The National Veterinary Services Laboratories (Ames, Iowa) obtained these cell lines in 1984 and froze the isolates.

**TABLE 1:** Listing of *Burkholderia mallei* isolate genomes released to NCBI

Strain	Accession no.	SRA	Source	Genome size (bp)	No. of contigs (scaffolds)	% G+C	No. of CDSs^*a*^	No. of tRNAs	No. of rRNAs
Turkey1	JNUY00000000	SRP060201	Horse	5,455,153	244 (2)	68.60	5,050	54	4
Turkey2	JNUZ00000000	SRP060209	Horse	5,475,037	302 (15)	68.60	5,135	51	5
Turkey4	JPGF00000000	SRP060248	Human	5,456,585	297 (5)	68.50	5,124	51	4
Turkey5	JPGI00000000	SRP060264	Human	5,421,535	239 (2)	68.50	5,095	50	4
Turkey6	JPGH00000000	SRP060249	Horse	5,446,114	229 (3)	68.50	5,045	55	5
Turkey7	JPGO00000000	SRP060250	Horse	5,655,761	141 (3)	68.50	5,058	52	5
Turkey8	JPGJ00000000	SRP060262	Horse	5,575,849	12 (2)	68.50	4,894	53	7
Turkey10	JNOV00000000	SRP060210	Horse	5,425,647	100 (2)	68.60	4,800	52	5

a CDS, coding sequence.

High-quality genomic DNA was extracted from purified isolates using QIAgen Genome Tip-500. Specifically, 100-mL bacterial cultures were grown to stationary phase and nucleic acid extracted as per manufacturer’s recommendations. Draft genome assemblies included two data sets (specific data types and coverages are listed in the NCBI records). Turkey 10 strain had Illumina short- (ca. 300-bp) and long-insert (generally >8 kb) paired data; all the other strains had Illumina short-insert paired data and Roche 454 long-insert paired data. Short- and long-insert paired data were assembled together in both Newbler and Velvet, and computationally shredded into 1.5 kbp overlapping shreds. All data were additionally assembled together in Allpaths (5). Consensus sequences from Allpaths were computationally shredded into 10 kbp overlapping pieces. All shreds were integrated using Phrap. Possible misassemblies were corrected and repeat regions verified using in-house scripts and manual editing in Consed (6, 7). Each genome assembly was annotated using an Ergatis-based (8) workflow with minor manual curation. *Burkholderia mallei* Turkey 3 and 9 were also sequenced, however, produced lower quality assemblies; these genomes can be found in NCBI under accession numbers JOLB00000000 and JNLU00000000, respectively.

Genome assemblies ranged from 12 to 302 contigs and 2 to 15 scaffolds (Table 1). As expected for the genus, % G+C was high, averaging 68.5%.

### Nucleotide sequence accession numbers.

Accession numbers for all 8 genomes are listed in Table 1.
